# A rare case of peritoneal tuberculosis mimicking peritoneal carcinomatosis: the ongoing challenge

**DOI:** 10.1186/s12879-024-10028-5

**Published:** 2024-10-10

**Authors:** Roberto M. Flores, Meena Dopp, Vivek V. Bhatt, Bassam Theodory, Paul M. Robb

**Affiliations:** 1 University of California, Riverside School of Medicine, Inland Empire, CA USA; 2 Department of Internal Medicine, Kaiser Permanente, 9961 Sierra Ave, Fontana, Inland Empire, CA 92395 USA

**Keywords:** Peritoneal carcinomatosis, Peritoneal tuberculosis, Ovarian cancer, CA-125, Case report

## Abstract

Tuberculosis (TB) is a serious infection that can involve any organ system and present in various forms. About one-third of the world’s population are carriers of latent TB. Although most cases are from a pulmonary origin, there is a rising prevalence of abdominal TB. Patients with pulmonary or extrapulmonary TB are treated similarly through the use of pharmacological therapy. Nonspecific clinical manifestations of TB have made it difficult for clinicians to diagnose. Peritoneal tuberculosis (PTB) is a serious concern as its symptoms overlap with that of many other chronic conditions, especially in those who are immunocompromised. The lack of highly sensitive and specific testing methods has made early intervention difficult, therefore a high index of suspicion is crucial in the progression of the disease. Here, we present a case of a 71-year-old female with a history of abdominal pain, fever, and weakness. Initial investigation with computed tomography (CT) imaging revealed omental fat stranding that pointed towards peritoneal carcinomatosis (PC) from possible recurrence of her ovarian cancer. Further investigation with a peritoneal biopsy was remarkable for caseating granulomas with fat necrosis confirming extrapulmonary TB. This report highlights a rare case of PTB mimicking PC in an elderly patient who is immunocompromised from the use of long-term corticosteroids who continued to decline after pharmacological treatment of the disease.

## Background

PTB is an uncommon form of extrapulmonary TB that presents with nonspecific clinical symptoms and commonly develops as a result of the reactivation of latent peritoneal foci [1, 2]. Extrapulmonary TB constitutes 18% of all tuberculosis cases in the United States, and PTB accounts for only 4% of all extrapulmonary disease [3, 4]. Liver cirrhosis, diabetes mellitus, Human Immunodeficiency Virus (HIV), prolonged corticosteroid use, malnutrition and end-stage renal disease on continuous ambulatory peritoneal dialysis have shown to be significant risk factors for PTB [5]. The diagnosis of PTB can be challenging as the clinical and radiological presentation of the condition can mirror malignancies, such as ovarian cancer or PC. Early diagnosis and treatment are crucial and no single test can effectively rule out this condition. Laparoscopy with peritoneal biopsy and subsequent histological findings of caseating granulomas remains to be a valuable approach for diagnosis [1, 6]. Regardless of history or risk factors, a differential diagnosis of patients who present with abdominal pain, ascites, and elevated Cancer Antigen 125 (CA-125) should include PTB [7, 8]. We describe a 71-year-old Vietnamese female on hydrocortisone 2.5 mg (taken with meals for the past year) for adrenal insufficiency and multiple comorbidities with abdominal pain, fever, cough and mild ascites that was found to have omental fat stranding of the anterior mesentery on CT imaging.

## Case presentation

A 71-year-old Vietnamese female with a significant past medical history of ovarian cancer (status post-TAH/BSO, tumor size 4 cm, Depth of Invasion 3/15 mm, Grade 1, negative for Lymphovascular Space Invasion, no history of chemotherapy use), adrenal insufficiency on chronic hydrocortisone, diabetes mellitus type 2, hypertension, and chronic kidney disease (stage 3) presented to the emergency department with severe weakness, abdominal pain, cough, fever, and poor oral intake. The patient was recently hospitalized with Coronavirus disease (COVID) pneumonia and treated with Remdesivir. The patient was noted to have an intra-abdominal fluid collection — following interventional radiology (IR) drainage, cultures were negative and suspected to be phlegmon. Repeat imaging showed resolution of the fluid collection and carcinomatosis was likely secondary to recurrence of ovarian cancer with labs significant for persistent leukocytosis and elevated CA-125. The patient was discharged with instructions to follow up with her primary care physician and oncologist for further management. At baseline, the patient was normally ambulatory with a front-wheel walker but was bed bound since discharge due to continued decline.

The patient returned four days after being discharged and upon arrival to the emergency department (ED), vital signs of the patient were 100.3 F body temperature, 86/70 mmHg, 101/min heart rate, 32/min respiratory rate and oxygen saturation of 99%. The patient’s blood pressure continued trending down, so she was given 2 L of 30 cc/kg. Laboratory tests were remarkable with a WBC at 21.0 mcL, Hgb at 10.6 g/dL, low potassium at 3.3 mEq/L, and alkaline phosphatase at 199 U/L. In the ED, urine studies were suggestive of urinary tract infection (UTI). Contrast-enhanced CT of abdomen and pelvis revealed mild ascites, moderate bilateral pleural effusions, generalized anasarca and fat stranding in the anterior mesentery on the left side representing possible metastasis with PC. The imaging workup was also suggestive of pneumonia, which was thought to be bacterial pneumonia secondary to recent COVID pneumonia, more likely hospital-acquired pneumonia vs. community acquired pneumonia. This prompted empiric broad-spectrum antibiotic treatment with vancomycin, cefepime, and metronidazole to cover hospital acquired pneumonia and potential UTI. Using these three antibiotics provides coverage against a wide range of bacterial pathogens, including gram-positive, gram-negative, and anaerobic bacteria. The start and end times for the administration of antibiotics for this patient were as follows: The patient was on Vancomycin from day 2 to day 5 and from day 23 to day 27, Cefepime from day 2 to day 7 and from day 23 to day 26, and Metronidazole from day 3 to day 7 and from day 24 to day 26.

**Fig. 1:**
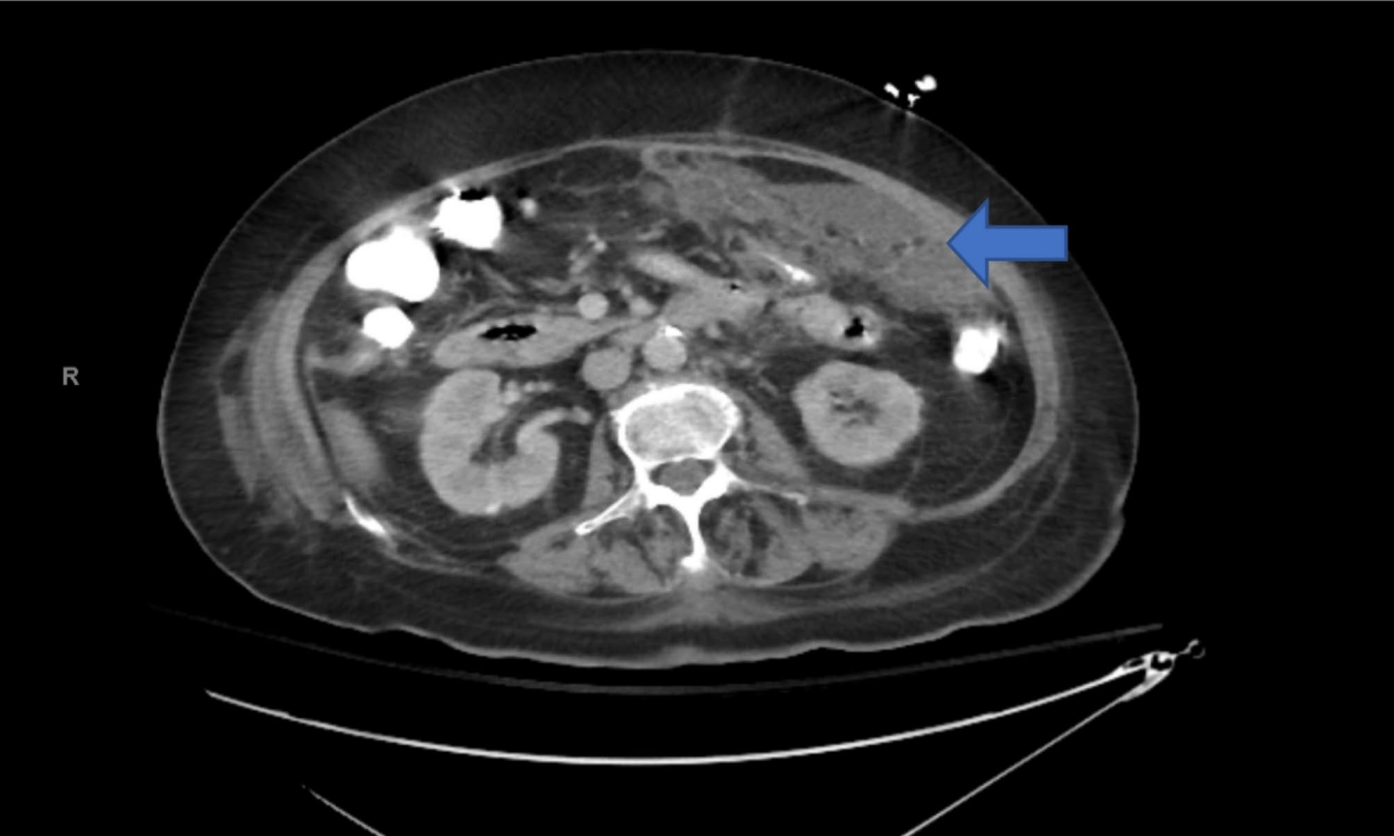
Axial CT section in a 71-year-old man with PTB revealing omental inflammation of the left peritoneum (indicated by the arrow)

On day 1 of admission, pertinent examination findings were diminished breath sounds, inspiratory crackles at lung bases, diffuse abdominal edema, tenderness in lower abdomen without guarding or rebound, and bilateral lower extremity edema. On initial assessment with bedside ultrasound, B-lines consistent with pulmonary edema and small pleural effusions were noted, but not large enough to warrant a thoracocentesis. The etiology of the pleural effusion was thought to be from the PC. Oncology recommended a CA-125 level and obtaining either ascites or pleural fluid to confirm the recurrence of cancer. Pleural fluid was not adequate enough to drain, so IR proceeded with a tissue biopsy of the omental fat stranding noted on imaging (see Fig. 1). Tumor markers were remarkable for CA-125 at 306.8 (normal < 38 u/mL). Previous admission had CA-125 at 436.3, CA 19-9  at 77 (normal < 37 U/mL) and carcinoembryonic antigen (CEA) at 10.9 (normal < 2.5 ng/mL).

**Fig. 2:**
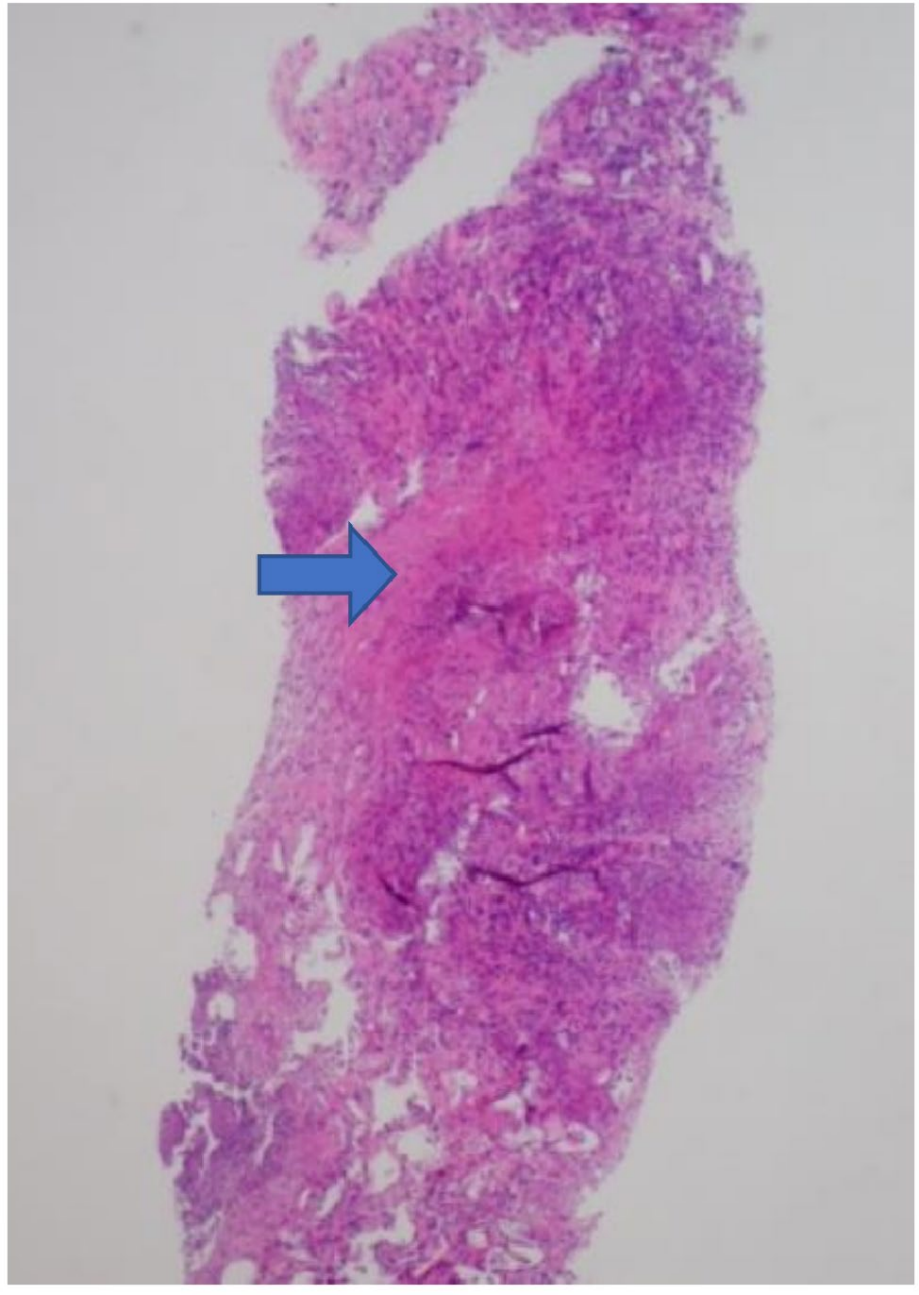
Histological finding of caseating granulomatous inflammation on the omentum (indicated by the arrow)

Other relevant test results from this patient were an albumin level of 3.0 g/dL in the peritoneal fluid, a cell count with differential showing red blood cells (RBC) at < 500 cells/mm³, nucleated cells at 676 cells/mm³, neutrophils at 11%, and lymphocytes at 55%. On day 5, a biopsy of the left revealed caseating granulomatous inflammation with fat necrosis (see Fig. 2). Despite the patient’s progressive decline, the etiology remained unclear, especially given the negative AFB culture preliminary results and negative PPD skin tests. No immediate PCR for M. Tuberculosis was deemed necessary at this time. According to the infectious disease consult, potential causes included tuberculosis, another mycobacterial disease, or potentially carcinomatosis, as indicated by the significantly elevated levels of CA-125 , CEA, and CA 19-9 . However, the initial biopsy did not reveal any malignancy in that specimen.

An AFB (Acid-Fast Bacilli) sputum culture is a laboratory test that involves culturing a patient’s sputum sample to detect the presence of acid-fast bacilli, particularly Mycobacterium tuberculosis, which causes tuberculosis. In the case of this patient, the Purified Protein Derivative (PPD) skin test and initial Acid-Fast Bacillus (AFB) sputum cultures (conducted three times) were negative. Grocott’s Methemenamine Silver (GMS) stain was negative for fungal organisms. The Interferon-gamma release assay (IGRA) and AFB stain of the biopsy yielded positive results. However, initially, there was low suspicion of abdominal TB due to the patient’s other co-morbidities and pending tests. A CT-guided paracentesis was performed with ascitic fluid studies for bacterial, fungal, AFB cultures, and cytology. Non-cytology studies found no evidence of recurrence of cancer.

On day 19, as the etiology remained unclear, a repeat IR CT-guided biopsy and paracentesis were performed. These tests detected a Mycobacterium Tuberculosis (MTB) PCR-positive complex using a Nucleic Acid Amplification Test (NAAT) and revealed caseating granulomas, leading to the diagnosis of extrapulmonary TB. The patient was started on empiric antituberculosis therapy (Rifampin, Isoniazid, Pyrazinamide, and Ethambutol) from day 21 to day 40. On day 26, the patient had a repeat AFB sputum culture that resulted as positive. A thoracocentesis was done the following day showing exudative effusion with a negative infectious workup. On day 32, a chest X-ray (CXR) revealed haziness or infiltrates in both lung bases. These findings had significantly improved compared to the previous study, with minimal pleural effusion noted in the left base. The patient had ongoing complications related to altered mentation, poor oral intake limiting medication intake, Catheter-associated urinary tract infection (CAUTI), urinary retention, gout with hyperuricemia secondary to pyrazinamide, anasarca, and subclinical hypothyroidism. The patient continued to decline with a developing fever, severe rigors, and unstable vital signs. A Pan-CT scan showed a larger fluid collection in the abdomen. The patient was less responsive, arterial blood gas showed hypercarbia, and Bilevel positive airway pressure (BiPAP) was started. On day 40, the patient was pronounced deceased due to severe sepsis with multiorgan failure.

## Discussion

PTB is a very rare disease with nonspecific symptoms that make it challenging to differentiate from other malignancies. This disease accounts for 0.1–0.7% of all tuberculosis cases worldwide [8]. Certain regions, especially in Vietnam, the patient’s country of origin, demonstrate a high incidence of tuberculosis. The patient immigrated to the United States from Vietnam in 1986. In 2020, Vietnam reported 172,000 cases of TB, ranking 10th globally among countries with the highest burdens of the disease [9]. The most common clinical sign is ascites, but patients may also present with abdominal pain, night sweats, fever, and abdominal distention [7]. The patient presented with complaints of abdominal pain, cough and fever, but it wasn’t enough to make the diagnosis. When corticosteroids are administered for treatment of other disease states, there’s a great risk of reactivating latent TB infection due to alteration of the cell-mediated immunity [2]. The patient’s long-term treatment of adrenal insufficiency with hydrocortisone likely contributed to the reactivation of latent TB.

The tumor marker CA-125 is elevated in several non-ovarian malignancies and generally, irritation of the pelvic or abdominal cavities is associated with an increased level [10]. This suggests that CA-125 is a non-specific marker for ovarian carcinoma and elevations have been reported in cases of patients presenting with PTB. An elevated CA-125 was seen in this patient, but as mentioned before, it can be secondary to the patient’s known history of ovarian cancer. It should be noted that treatment with quadruple anti-tubercular drugs has shown to gradually decrease the level of CA-125 and reaches a normal range after 1 or 2 months [11, 12]. This serves as another way to confirm the diagnosis of PTB.

The differentiation between PC and PTB on imaging is challenging due to many overlapping findings [11]. Contrast-enhanced imaging of the abdomen and pelvis of this patient revealed ill-defined peritoneal thickening and fatty stranding, which is a similar feature of both diagnoses. A (CXR) was also negative for primary pulmonary tuberculosis, which indicated that reactivation of latent TB did not result from hematogenous spread from a primary lung focus [1]. Prior studies have indicated that primary pulmonary tuberculosis on CXR imaging is only seen in 15–20% of patients with PTB [8]. 

A positive tuberculin skin test (TST) or interferon-gamma release assay (IGRA) can indicate TB infection but cannot distinguish between latent and active TB. Moreover, a negative TST or IGRA does not rule out the diagnosis of active TB, as patients with histologically confirmed peritoneal TB may still present with negative results [13]. A negative result doesn’t exclude the condition, and this was seen with the patient who had a negative PPD test, but a histological finding remarkable for caseating granulomas. It’s important to note that a negative TST can be attributed to the patient’s immunosuppression, which may impair the immune response required for a positive test. A delayed diagnosis of peritoneal TB may also be a result of the high false negative rates of AFB smears and AFB cultures taking between 4 and 8 weeks to grow [14]. Three AFB cultures were collected and one AFB sputum culture a few weeks before the patient’s death presented as positive. Measurements of gamma interferon (IFN-y) have been used to diagnose peritoneal TB with high sensitivity and specificity, but this noninvasive test doesn’t replace the peritoneal biopsy [6, 7, 13]. The patient had a positive gamma interferon test, but the other negative laboratory results made the diagnosis challenging. The delay in initiating TB treatment due to this diagnostic difficulty may have adversely affected the patient’s outcome, potentially leading to prolonged illness, increased risk of complications, and a delay in recovery.

A repeat peritoneal biopsy and paracentesis was remarkable for caseating granulomas with MTB PCR positive. This led to the diagnosis of PTB and initiation of quadruple anti-tubercular therapy. The hospital course was complicated by lethargy, poor oral intake and around the time of diagnosis, the patient continued to decline until pronounced deceased due to severe sepsis with multiorgan failure. Patients who go undiagnosed and therefore untreated with PTB may lead to severe sepsis, advanced tuberculosis, and death [4]. Initially, there was high suspicion of the patient having PC due to omental inflammation seen on CT and their known history of ovarian cancer. An elevated CA-125, nonspecific symptoms, and several negative laboratory results made the diagnosis unclear. The obscure etiology of this patient’s suffering led to a delay in treatment, which led to progression of disease that was no longer responsive to pharmacological intervention.

Although the patient was eventually diagnosed with this condition, there was no clinical improvement with pharmacological therapy. Our search of literature revealed that other cases with peritoneal TB improved with quadruple therapy, but this patient continued to decline [7]. Besides delayed intervention, the clinical decline could have also been attributed to the patient’s age, disease progression and several comorbidities. The age at which PTB peaks is ages 20–40, which is an earlier diagnosis than that seen in ovarian or PC [4]. The patient’s elderly age is what also makes this case report different from others. This is a rare case of PTB mimicking PC, and it highlights the importance of early diagnosis and disease progression. Thus, we present an elderly female with omental inflammation on CT imaging mimicking PC that was ruled out by histological examination through a CT-guided biopsy of the peritoneum, confirming PTB.

## Data Availability

All data generated or analyzed during this study are included in this published article.

## Abbreviations

TB: Tuberculosis
PTB: Peritoneal Tuberculosis
PC: Peritoneal Carcinomatosis
HIV: Human Immunodeficiency Virus
CA-125: Cancer Antigen 125
CT: Computed tomography
TAH: Total Abdominal Hysterectomy
BSO: Bilateral Salpingo-Oophorectomy
CXR: Chest X-ray
Bipap: Bilevel positive airway pressure
CAUTI: Catheter-associated urinary tract infection
GMS: Grocott’s Methemenamine Silver
AFB: Acid Fast Bacillus
PPD: Purified Protein Derivative
CEA: Carcinoembryonic antigen
UTI: Urinary tract infection
MTB: Mycobacterium tuberculosis
